# Author Correction: Hypoxia induces histone clipping and H3K4me3 loss in neutrophil progenitors resulting in long-term impairment of neutrophil immunity

**DOI:** 10.1038/s41590-025-02380-8

**Published:** 2025-12-01

**Authors:** Manuel A. Sanchez-Garcia, Pranvera Sadiku, Brian M. Ortmann, Niek Wit, Yutaka Negishi, Patricia Coelho, Ailiang Zhang, Chinmayi Pednekar, Andrew J. M. Howden, David M. Griffith, Rachel Seear, Jessica D. Kindrick, Janine Mengede, George Cooper, Tyler Morrison, Emily R. Watts, Benjamin T. Shimeld, Leila Reyes, Ananda S. Mirchandani, Simone Arienti, Xiang Xu, Alexander Thomson, Alejandro J. Brenes, Helena A. Turton, Rebecca Dowey, Rebecca C. Hull, Hazel Davidson-Smith, Amy McLaren, Andrew Deans, Gourab Choudhury, Katherine Doverman, David Hope, Oliver Vick, Alastair Woodhead, Isla Petrie, Suzanne Green, Nina M. Rzechorzek, Lance Turtle, Peter J. M. Openshaw, Malcolm G. Semple, the PHOSP-COVID Study Collaborative Group, Duncan Sproul, J. Kenneth Baillie, Alfred A. R. Thompson, David R. Mole, Alex von Kriegsheim, Moira K. B. Whyte, Musa M. Mhlanga, James A. Nathan, Sarah R. Walmsley

**Affiliations:** 1 Centre for Inflammation Research, Institute for Regeneration and Repair, University of Edinburgh, Edinburgh, UK; 2 Cambridge Institute of Therapeutic Immunology and Infectious Disease (CITIID), Jeffrey Cheah Biomedical Centre, Department of Medicine, University of Cambridge, Cambridge, UK; 3 Department of Internal Medicine and Radboud Center for Infectious Diseases, Radboud University Medical Center, Nijmegen, the Netherlands; 4 Department of Cell Biology, Faculty of Science, Radboud Institute for Molecular Life Sciences, Radboud University Nijmegen, Nijmegen, the Netherlands; 5 Department of Human Genetics, Radboud University Medical Center, Nijmegen, the Netherlands; 6 CRUK Scotland Centre, IGC, University of Edinburgh, Edinburgh, UK; 7 Division of Cell Signalling and Immunology, University of Dundee, Dundee, UK; 8 The Usher Institute, University of Edinburgh, Royal Infirmary of Edinburgh, Edinburgh, UK; 9 NDM Research Building, Nuffield Department of Medicine, University of Oxford, Oxford, UK; 10 Centre for Gene Regulation and Expression, University of Dundee, Dundee, UK; 11 Division of Clinical Medicine, University of Sheffield, Sheffield, UK; 12 MRC Human Genetics Unit, MRC IGC, University of Edinburgh, Edinburgh, UK; 13 NHS Lothian, Respiratory Medicine, Edinburgh, UK; 14 Edinburgh Critical Care Research Group, NHS Lothian, Royal Infirmary of Edinburgh, Edinburgh, UK; 15 Altitude Physiology Expeditions (APEX), Sheffield, UK; 16 MRC Laboratory of Molecular Biology, Cambridge, UK; 17 Department of Engineering, University of Cambridge, Cambridge, UK; 18 Health Protection Research Unit in Emerging and Zoonotic Infections, Institute of Infection, Veterinary and Ecological Sciences, University of Liverpool, Liverpool, UK; 19 Tropical and Infectious Disease Unit, Liverpool University Hospitals NHS Foundation Trust (member of Liverpool Health Partners), Liverpool, UK; 20 Respiratory Infection Section, National Heart and Lung Institute, Imperial College London, London, UK; 21 Respiratory Department, Liverpool Institute of Child Health and Wellbeing, Alder Hey Children’s Hospital NHS Foundation Trust, Liverpool, UK; 22 MRC Human Genetics Unit and CRUK Edinburgh Centre, MRC IGC, University of Edinburgh, Edinburgh, UK; 23 The Roslin Institute, University of Edinburgh, Edinburgh, UK

**Keywords:** Neutrophils, Chronic inflammation

Correction to: *Nature Immunology* 10.1038/s41590-025-02301-9, published online 28 October 2025.

In the version of this article initially published, the surname of Musa M. Mhlanga was misspelled (Mhalanga) and is now amended in the HTML and PDF versions of the article.

