# Supplementary material for: Hypoxia induces histone clipping and H3K4me3 loss in neutrophil progenitors resulting in long-term impairment of neutrophil immunity
Source: Nat Immunol. 2025 Oct 28;26(11):1903–15. doi: 10.1038/s41590-025-02301-9 (PMC12571872; doi:10.1038/s41590-025-02301-9)
Supplement: Supplementary file 1 — Supplementary Table 1. [file 41590_2025_2301_MOESM1_ESM.pdf]

nature immunology

Supplementary information https://doi.org/10.1038/s41590-025-02301-9

Hypoxia induces histone clipping and H3K4me3 loss in neutrophil progenitors resulting in long-term impairment of neutrophil immunity

In the format provided by the authors and unedited

Supplementary Table 1: ARDS survivors patient cohort clinical characteristics and demographics.

Number of patients	52
Age (years) – mean ± s.d.	55.4 ± 11.6
Proportion of females – number (%)	20 (38)
Body mass index (kg/m^2) – mean ± s.d.	32.2 ± 7.8
APACHE II[a] Score[b] – mean ± s.d.	16.3 ± 6.7
Aetiologies of ARDS	
Pulmonary – number (%)	48 (92)
Positive viral PCR[c] – number	39
Positive bacterial culture – number	6
Positive mycology – number	1
Positive microbiology samples – number	2
Extra-pulmonary – number (%)	4 (8)
Pancreatitis[d] – number	3
Bacteraemia – number	1
Features of acute inflammation	
Reference white cell count ($x10^9/L$)[e]	4.0-11.0
White cell count at recruitment ($x10^9/L$) – mean ± s.d.	10.46 ± 4.53
Reference neutrophil count ($x10^9/L$)[e]	2.0-7.5
Neutrophil count at recruitment ($x10^9/L$) – mean ± s.d.	8.48 ± 4.32
Predictors of tissue hypoxia	
Reference partial pressure of arterial oxygen (PaO_2, KPa)[e]	11.1-14.4
Lowest PaO_2 in hospitalisation before recruitment (KPa) – mean ± s.d.	5.75 ± 1.91
Lowest PaO_2 24 h preceding recruitment (KPa) – mean ± s.d.	7.74 ± 1.08
Reference fraction of inspired oxygen (FiO_2)[e] – %	21
Highest FiO_2 in 24 h before recruitment – mean ± s.d.	68.9 ± 22.8
Reference lactate (mmol/L)[e] – mean ± s.d.	0.5-1.6
Highest lactate 24 h before recruitment (mmol/L) – mean ± s.d.	1.53 ± 0.75
Receipt of organ supportive therapies – number (%)	
Invasive mechanical ventilation	24 (46)
High flow nasal oxygen	25 (48)
Non-invasive ventilation	3 (6)
Vasopressors	14 (27)
Renal replacement therapy	2 (4)
Receipt of additional medications – number (%)	
Dexamethasone[f]	29 (54)
Lopinavir and ritonavir	1 (2)
Tocilizumab	12 (23)
Hydroxychloroquine	2 (4)

[a]Acute Physiology and Chronic Health Evaluation Score II (APACHE II). [b]APACHE II score only available for 24 patients. [c]Thirty-nine patients were diagnosed with acute respiratory distress syndrome (ARDS) secondary to PCR-confirmed COVID-19 pneumonitis. [d]Three patients were diagnosed with ARDS secondary to pancreatitis, two of which were bacterial infections, with a single case of necrotising alcohol-induced pancreatitis. [e]Reference ranges as indicated by local health board (NHS Lothian). [f]No other corticosteroids were administered.
