# Supplementary material for: Hypoxia induces histone clipping and H3K4me3 loss in neutrophil progenitors resulting in long-term impairment of neutrophil immunity
Source: Nat Immunol. 2025 Oct 28;26(11):1903–15. doi: 10.1038/s41590-025-02301-9 (PMC12571872; doi:10.1038/s41590-025-02301-9)
Supplement: Supplementary file 2 — Reporting Summary [file 41590_2025_2301_MOESM2_ESM.pdf]

nature portfolio

Corresponding author(s): Sarah R. Walmsley

Last updated by author(s): Aug 27, 2025

Reporting Summary

Nature Portfolio wishes to improve the reproducibility of the work that we publish. This form provides structure for consistency and transparency in reporting. For further information on Nature Portfolio policies, see our Editorial Policies and the Editorial Policy Checklist.

Statistics

For all statistical analyses, confirm that the following items are present in the figure legend, table legend, main text, or Methods section.

n/a	Confirmed	
☐	☒	The exact sample size (*n*) for each experimental group/condition, given as a discrete number and unit of measurement
☐	☒	A statement on whether measurements were taken from distinct samples or whether the same sample was measured repeatedly
☐	☒	The statistical test(s) used AND whether they are one- or two-sided<br>*Only common tests should be described solely by name; describe more complex techniques in the Methods section.*
☒	☐	A description of all covariates tested
☐	☒	A description of any assumptions or corrections, such as tests of normality and adjustment for multiple comparisons
☐	☒	A full description of the statistical parameters including central tendency (e.g. means) or other basic estimates (e.g. regression coefficient) AND variation (e.g. standard deviation) or associated estimates of uncertainty (e.g. confidence intervals)
☐	☒	For null hypothesis testing, the test statistic (e.g. F, t, r) with confidence intervals, effect sizes, degrees of freedom and P value noted<br>*Give P values as exact values whenever suitable.*
☒	☐	For Bayesian analysis, information on the choice of priors and Markov chain Monte Carlo settings
☒	☐	For hierarchical and complex designs, identification of the appropriate level for tests and full reporting of outcomes
☒	☐	Estimates of effect sizes (e.g. Cohen's *d*, Pearson's *r*), indicating how they were calculated

Our web collection on statistics for biologists contains articles on many of the points above.

Software and code

Policy information about availability of computer code

Data collection	Custom scripts generated have been deposited in Zenodo. Details for access included in the manuscript.
Data analysis	Proteomics data was analysed with Spectronaut 14 and 16 using the directDIA option.<br>PCA plots were generated with Perseus software.<br>Metabolomics analysis was performed with Skyline software (21.1).<br>ChIPseq data was processed and analysed by using Trim Galore (0.6.7), HISAT (2.2.1), bedtools (2.30), samtools (1.15), Picard (3.1.0), ngs.plot (2.61), MACS3 (3.0.0.a6), custom scripts of the R packages DiffBind, ChIPseeker, and ReactomePA, HOMER (4.11), and a custom Python 3.10.8 script.<br>Cut&Run data was generated aligning trimmed reads with BWA and peaks called by MACS2.<br>FlowJo (10.8.1) was used to analyse flow cytometry data.<br>Data was analysed with Graphpad prism (10.5.0).

For manuscripts utilizing custom algorithms or software that are central to the research but not yet described in published literature, software must be made available to editors and reviewers. We strongly encourage code deposition in a community repository (e.g. GitHub). See the Nature Portfolio guidelines for submitting code & software for further information.

Data

Policy information about availability of data

All manuscripts must include a data availability statement. This statement should provide the following information, where applicable:

- Accession codes, unique identifiers, or web links for publicly available datasets
- A description of any restrictions on data availability
- For clinical datasets or third party data, please ensure that the statement adheres to our policy

Proteomic data from ARDS survivors, high-altitude study volunteers and preNeu has been deposited in PRIDE and accession codes are provided. ChiPseq and Cut&Run data have been uploaded to GEO and accession codes are provided. Accession tokens have been generated and are readily available.

Research involving human participants, their data, or biological material

Policy information about studies with human participants or human data. See also policy information about sex, gender (identity/presentation), and sexual orientation and race, ethnicity and racism.

Reporting on sex and gender	Human data from both sexes were included in this study and recorded by the clinical teams upon hospital visit or as self-reported by the participant. No sex stratification studies have been performed. The acute respiratory disease syndrome patient cohort included 52 individuals, with a male:female ratio of 32:20. The altitude induced hypoxemia cohort included 20 participants, with a male:female ratio of 8:12. The healthy donors used as control group was integrated by 33 participants, with a male:female ratio of 10:23. We have ethical approval to show individual anonymized data.
Reporting on race, ethnicity, or other socially relevant groupings	We have not performed any analysis based on race, ethnicity or any other socially relevant grouping.
Population characteristics	The population characteristics for the acute respiratory disease syndrome patient cohort are collated in Table 1. The average age of the participants involved in the altitude induced hypoxia study was 22 years old. The average age range of the healthy donors used as controls was 31-40 years old. No extra characteristics were allowed to be recorded from either the altitude induced hypoxia or the control cohorts.
Recruitment	Patient recruitment took place from April 2020-April 2025 mainly at The Royal Infirmary of Edinburgh, UK, the University of Cambridge and the University of Sheffield. The acute respiratory distress syndrome patients included in this study were males and females diagnosed according to the Berlin criteria. All sequential samples from patients identified based on the ethics inclusion criteria were processed. Survivors were samples 3-6 months post-hospital admission. This study was performed with informed consent.<br>The altitude induced hypoxemia cohort was restricted to participants who had voluntarily enrolled in the Apex6 expedition and written consent was taken from all of them.
Ethics oversight	Acute respiratory distress syndrome studies were done through the ARDS Neut study (20/SS/0002) and in association with the PHOSP study (20/YH/0225), the University of Cambridge (17/EE/0025) and the University of Sheffield (18/YH/0441).<br>The altitude induced hypoxia study was approved by the Edinburgh Medical School Research Ethics Committee (EMREC, 21-EMREC-043).<br>The healthy donors blood donations were regulated by the University of Edinburgh Centre for Inflammation Research Blood Resource Management Committee (AMREC 15-HV-013, 21-EMREC-041).

Note that full information on the approval of the study protocol must also be provided in the manuscript.

Field-specific reporting

Please select the one below that is the best fit for your research. If you are not sure, read the appropriate sections before making your selection.

☒ Life sciences ☐ Behavioural & social sciences ☐ Ecological, evolutionary & environmental sciences

For a reference copy of the document with all sections, see nature.com/documents/nr-reporting-summary-flat.pdf

Life sciences study design

All studies must disclose on these points even when the disclosure is negative.

Sample size	Groups of at least 3 biological replicates were used in each experiment in each treatment or experimental group. Data from individual replicate experiments were pooled as detailed in the figure legends. No sample calculations were performed pre-hoc. Sample size was based on pilot experiments and previous experience with similar experiments in the laboratory.
Data exclusions	One of the mice in the experiment detailed in Figure 5b-c was not included in the analysis as did not develop infection at all.
Replication	Experiments were replicated and pooled as detailed in the figure legends.

Randomization	For human studies, due to limited sample volumes, cell availability and high demands on cell number for "omics assays", it was not possible to conduct every assay on each patient sampled. Assays were performed sequentially as samples became available/cell number permitting with no prior selection.<br>In mouse studies, mice were randomly allocated to normoxic and hypoxic groups. Mixing of cages was not permitted where male mice were used but were purchased as part of the same cohort. Mice were age- and sex-matched within and between experiments.
Blinding	Experimental processing, data acquisition, and data analysis were performed on a blinded way whenever possible.

Reporting for specific materials, systems and methods

We require information from authors about some types of materials, experimental systems and methods used in many studies. Here, indicate whether each material, system or method listed is relevant to your study. If you are not sure if a list item applies to your research, read the appropriate section before selecting a response.

Materials & experimental systems

n/a	Involved in the study
☐	☒ Antibodies
☒	☐ Eukaryotic cell lines
☒	☐ Palaeontology and archaeology
☐	☒ Animals and other organisms
☒	☐ Clinical data
☒	☐ Dual use research of concern
☒	☐ Plants

Methods

n/a	Involved in the study
☐	☒ ChIP-seq
☐	☒ Flow cytometry
☒	☐ MRI-based neuroimaging

Antibodies

Antibodies used

CD16 eBioCB16 11-0168-42 2253005 FITC Ebioscience 1:200
CD66b G10F5 305114 B292150 AF700 Biolegend 1:200
CD62L DREG-56 304814 B291262 APC-Cy7 Biolegend 1:200
CD49d 9F10 304322 B302258 BV421 Biolegend 1:200
CD45 30-F11 103128 B380021 AF700 Biolegend 1:200
Ly6G 1A8 127628 B431781 BV421 Biolegend 1:200
Fc block 93 101320 B411434 Unconjugated Biolegend 1:100
H3K4me3 C42D8 9751S 15 Unconjugated Cell Signaling 1:50
H3K4me3 Polyclonal 07-473 2289139 Unconjugated Upstate 1:10000
Goat anti-rabbit Polyclonal 925-32211 D00804-06 IRDye 800CW Li-cor 1:10000
H4 Sp2/0-Ag14 Ab31830 GR3204774-3 Unconjugated Abcam 1:1000
Goat anti-mouse Polyclonal 925-68070 C90910-20 IRDye 680RD Li-cor 1:1000
CD16/32 93 101326 B418060 APC Biolegend 1:200
Lineage n/a 78022 B281150 FITC Biolegend 1:200
Sca1 D7 108129 B444060 BV510 Biolegend 1:100
c-Kit 2B8 105835 B249350 BV711 Biolegend 1:200
CD34
SA376A4 152208 B419071 BV421 Biolegend 1:200
CD16/32 93 101333 B418160 BV510 Biolegend 1:100
CD90.2 53-2.1 13-0902-82 3020994 Biotin Ebioscience 1:200
B220 (CD45R) RA3-6B2 13-0452-82 2899900 Biotin Ebioscience 1:200
NK1.1 PK136 13-5941-82 2791123 Biotin Ebioscience 1:200
Sca1 D7 13-5981-82 2993028 Biotin Ebioscience 1:200
Flt3 A2F10 13-1351-82 2924264 Biotin Ebioscience 1:200
CD115 AFS98 13-1152-82 2986854 Biotin Ebioscience 1:200
Ter119 TER-119 13-5921-82 2609055 Biotin Ebioscience 1:200
CD34
SA376A4 152218 B438235 PE-Cy7 Biolegend 1:100
Ly6G 1A8 127628 B431781 BV421 Biolegend 1:200
CD11b M1/70 101257 B431815 BV605 Biolegend 1:200
c-Kit 2B8 105835 B429186 BV711 Biolegend 1:200
CD106 429 105716 B399866 PerCP-Cy5.5 Biolegend 1:200
SiglecF S17007L 155534 NB424840 AF700 Biolegend 1:200
Ly6C Hk1.4 128041 B406937 BV785 Biolegend 1:500
Gr1 RB6-8C5 108417 B410115 AF488 Biolegend 1:500
H3 EPR17785 ab313347 1076340-2 AF647 Abcam 1:500
H3K4me3 EPR20551-225 ab237342 1027184-1 PE Abcam 1:1000

Validation

All the antibodies were used according to manufacturer's guidelines.
CD16 11-0168-42 Ebioscience Santana-Hernandez S, et al. 2024. J Exp Clin Cancer Res.
CD66b 305114 Biolegend Giamarellos-Bourboulis EJ, et al. 2020. Cell.

CD62L 304814 Biolegend Ajith A, et al. 2021. Front Immunol.
CD49d 304322 Biolegend Qi Q, et al. 2020. Blood.
CD45 103128 Biolegend Radtke AJ, et al. 2022. Nat Protoc.
Ly6G 127628 Biolegend Hutter K, et al. 2022. Front Immunol.
Fc block 101320 Biolegend Ajith A, et al. 2021. Front Immunol.
H3K4me3 9751S Cell Signaling Noshita et al. 2023. J Cell Sci.
H3K4me3 07-473 Upstate Suijker et al. 2015. Oncotarget
Goat anti-rabbit 925-32211 Li-cor This antibody was tested by dot blot and and/or solid-phase. The conjugate has been specifically tested and qualified for Western blot and In-Cell Western™ Assay applications.
H4 Ab31830 Abcam Samejima et al. 2022. Mol Cell.
Goat anti-mouse 925-68070 Li-cor This antibody was tested by dot blot and and/or solid-phase. The conjugate has been specifically tested and qualified for Western blot and In-Cell Western™ Assay applications.
CD16/32 101326 Biolegend Lopez DA, et al. 2022. Cell Rep.
Lineage 78022 Biolegend Mirchandani AS, et al. 2022. Nat Immunol.
Sca1 108129 Biolegend Sandovici I, et al. 2022. Dev Cell.
c-Kit 105835 Biolegend Schönberger K, et al. 2022. Cell Stem Cell
CD34 152208 Biolegend Vanneste D, et al. 2023. Nat Immunol.
CD16/32 101333 Biolegend Al-Rifai R, et al. 2022. Nat Commun.
CD90.2 13-0902-82 Ebioscience Bi R, et al. 2023. Nat Commun.
B220 (CD45R) 13-0452-82 Ebioscience Vos WG, et al. 2024. Front. Immunol.
NK1.1 13-5941-82 Ebioscience Chen Y, et al. 2024. Cell Commun Signal.
Sca1 13-5981-82 Ebioscience Griffin KH, et al. 2023. PNAS.
Flt3 13-1351-82 Ebioscience Qiu J, et al. 2024. Stemm Cell Res Ther.
CD115 13-1152-82 Ebioscience Eren RO, et al. 2024. Nat Commun.
Ter119 13-5921-82 Ebioscience Tundidor I, et al. 2023. Nat Commun.
CD34 152218 Biolegend Biolegend's QC testing. Xu Y, et al. 2023. EMBO Rep. for same clone ref.
Ly6G 127628 Biolegend Hutter K, et al. 2022. Front Immunol.
CD11b 101257 Biolegend Gallizioli M, et al. 2020. Cell Rep.
c-Kit 105835 Biolegend Schönberger K, et al. 2022. Cell Stem Cell.
CD106 105716 Biolegend Nahrendorf W, et al. 2021. eLife.
SiglecF 155534 Biolegend Biolegend's QC testing. Senatus L, et al. 2023. Commun Biol. for same clone ref.
Ly6C 128041 Biolegend Grandjean CL, et al. 2021. Sci Adv.
Gr1 108417 Biolegend Haratani K, et al. 2019. J Clin Invest.
H3 ab313347 Abcam Abcam's flow cytometry testing service
H3K4me3 ab237342 Abcam Abcam's flow cytometry testing service

Animals and other research organisms

Policy information about studies involving animals; ARRIVE guidelines recommended for reporting animal research, and Sex and Gender in Research

Laboratory animals	Animal experiments were conducted in accordance with the UK Home Office Animals (Scientific Procedures) Act of 1986. All animal studies were approved by The University of Edinburgh Animal Welfare and Ethical Review Board, adhered to the principles of "3Rs" (replacement, reduction, refinement), and complied with ARRIVE guidelines for animal research. Mice of 3-6 months old were used for these studies. Mice were housed in IVC cages under 12 h light/darkness cycles and controlled temperature (20-23°C) in accordance with UK Home Office guidance. All mice used for experiments were healthy with quarterly and annual testing carried out in accordance with FELASA 2014 Guidelines, using a mixture of environmental, random colony samples and sentinel testing by serology and PCR. Mice had ad libitum access to food and water.
Wild animals	N/A
Reporting on sex	92 male mice were used in this study. According to our experience using LPS models and to the literature (Aziz et al. 2007, Mock et al. 2023), age and weight of the mice may have an impact on inflammatory outcomes but sex, per se, does not have a significant effect. Additionally, any changes in outcomes are in magnitude or duration rather than character. We therefore performed experiments only in male mice to avoid potential magnitude variability and reduce the number of animals needed for our studies, in keeping with the 3R perspective on experimental animal usage.
Field-collected samples	N/A
Ethics oversight	All animal studies were approved by The University of Edinburgh Animal Welfare and Ethical Review Board.

Note that full information on the approval of the study protocol must also be provided in the manuscript.

Plants

Seed stocks	N/A
Novel plant genotypes	N/A

Authentication | N/A

ChIP-seq

Data deposition

☒ Confirm that both raw and final processed data have been deposited in a public database such as GEO.

☒ Confirm that you have deposited or provided access to graph files (e.g. BED files) for the called peaks.

Data access links
May remain private before publication.

GSE240723

Files in database submission

H3K4me3: .bigwig, .bed, and .fastq files
2_H3K4me3-sort-bl-dedupl-downscaled-norm.bw
3_H3K4me3-sort-bl-dedupl-downscaled-norm.bw
4_H3K4me3-sort-bl-dedupl-downscaled-norm.bw
8_H3K4me3-sort-bl-dedupl-downscaled-norm.bw
9_H3K4me3-sort-bl-dedupl-downscaled-norm.bw
10_H3K4me3-sort-bl-dedupl-downscaled-norm.bw
11_H3K4me3-sort-bl-dedupl-downscaled-norm.bw
13_H3K4me3-sort-bl-dedupl-downscaled-norm.bw
14_H3K4me3-sort-bl-dedupl-downscaled-norm.bw
15_H3K4me3-sort-bl-dedupl-downscaled-norm.bw
2_H3K4me3-sort-bl-dedupl-downscaled_2.bam.bed
3_H3K4me3-sort-bl-dedupl-downscaled_3.bam.bed
4_H3K4me3-sort-bl-dedupl-downscaled_4.bam.bed
8_H3K4me3-sort-bl-dedupl-downscaled_3.bam.bed
9_H3K4me3-sort-bl-dedupl-downscaled_4.bam.bed
10_H3K4me3-sort-bl-dedupl-downscaled_5.bam.bed
11_H3K4me3-sort-bl-dedupl-downscaled_1.bam.bed
13_H3K4me3-sort-bl-dedupl-downscaled_3.bam.bed
14_H3K4me3-sort-bl-dedupl-downscaled_4.bam.bed
15_H3K4me3-sort-bl-dedupl-downscaled_5.bam.bed
WTCHG_917224_73305306_1.fastq.gz
WTCHG_917224_73305306_2.fastq.gz
WTCHG_917224_73315307_1.fastq.gz
WTCHG_917224_73315307_2.fastq.gz
WTCHG_917224_73325308_1.fastq.gz
WTCHG_917224_73325308_2.fastq.gz
WTCHG_917224_73365312_1.fastq.gz
WTCHG_917224_73365312_2.fastq.gz
WTCHG_917224_73375313_1.fastq.gz
WTCHG_917224_73375313_2.fastq.gz
WTCHG_917224_73385314_1.fastq.gz
WTCHG_917224_73385314_2.fastq.gz
WTCHG_917224_73395315_1.fastq.gz
WTCHG_917224_73395315_2.fastq.gz
WTCHG_917224_73415317_1.fastq.gz
WTCHG_917224_73415317_2.fastq.gz
WTCHG_917224_73425318_1.fastq.gz
WTCHG_917224_73425318_2.fastq.gz
WTCHG_917224_73435319_1.fastq.gz
WTCHG_917224_73435319_2.fastq.gz

Input controls: .fastq files
WTCHG_917224_73145290_1.fastq.gz
WTCHG_917224_73145290_2.fastq.gz
WTCHG_917224_73155291_1.fastq.gz
WTCHG_917224_73155291_2.fastq.gz
WTCHG_917224_73165292_1.fastq.gz
WTCHG_917224_73165292_2.fastq.gz
WTCHG_917224_73205296_1.fastq.gz
WTCHG_917224_73205296_2.fastq.gz
WTCHG_917224_73215297_1.fastq.gz
WTCHG_917224_73215297_2.fastq.gz
WTCHG_917224_73225298_1.fastq.gz
WTCHG_917224_73225298_2.fastq.gz
WTCHG_917224_73235299_1.fastq.gz
WTCHG_917224_73235299_2.fastq.gz
WTCHG_917224_73255301_1.fastq.gz
WTCHG_917224_73255301_2.fastq.gz
WTCHG_917224_73265302_1.fastq.gz
WTCHG_917224_73265302_2.fastq.gz

	WTCHG_917224_73275303_1.fastq.gz<br>WTCHG_917224_73275303_2.fastq.gz
Genome browser session (e.g. UCSC)	IGV

Methodology

Replicates	3 independent samples from healthy donors<br>3 independent samples from ARDS survivors 3-6 months post-hospital admission not treated with dexamethasone<br>4 independent samples from ARDS survivors 3-6 months post-hospital admission treated with dexamethasone
Sequencing depth	75-bp paired-end sequencing. HiSeq 4000 platform (Illumina)
Antibodies	Refer to Table 2.<br>H3K4me3 C42D8 9751S 15 Cell Signaling 1:50
Peak calling parameters	Raw sequencing reads were first quality trimmed using Trim Galore 0.6.7 using standard settings. Read mapping was performed with HISAT2 2.2.1 against the human genome (hg19) in paired-end mode. Reads mapping to blacklisted regions were removed with the bedtools 2.30 intersect tool and sorted by coordinate and indexed using samtools 1.15 sort and index, respectively. From the resulting BAM files, duplicated reads were removed using the MarkDuplicates tool from Picard with the setting REMOVE_DUPLICATES=TRUE. Any potential differences in sequencing depth were corrected by down-sampling all samples to the same number of fragments. Corresponding input files were used as controls. For each individual BAM file, H3K4me3 peaks were identified using the MACS3 3.0.0.a6 callpeak function with settings -q 0.05 -g hs -f BAMPE --broad --broad-cutoff 0.1.
Data quality	Successful sample fragmentation was confirmed by analysis with TapeStation (Agilent). MACS3 3.0.0.a6 callpeak function was used with settings -q 0.05 -g hs -f BAMPE --broad --broad-cutoff 0.1. Differential H3K4me3 peak analysis between conditions, and generation of the H3K4 signal heatmap, PCA plot, sample distance heatmap and enriched pathway analysis of differential peaks was performed with R 4.2.0 using the Bioconductor packages DiffBind, ChIPseeker, clusterProfiler, and ReactomePA. Any potential differences in sequencing depth were corrected by down-sampling all samples to the same number of fragments.<br>BAM files were merged per group with samtools merge to create subsequent metagene H3K4me3 profiles with ngs.plot 2.6163. This software performs 2 normalization steps: the coverage vectors (i. e. gene regions) are normalized to be equal length, and the vectors are normalized against the corresponding library size (i. e. the total read count for the reads that pass quality filters) to generate the Reads Per Million (RPM) mapped reads. Additionally, data from each individual was normalized by its corresponding input sample. We represented 10000 bins around transcription start sites, which confirmed a higher concentration of peaks at these genomic areas across study groups, as expected according to previous studies. The random distribution of 10000 bins along the DNA revealed equivalent low abundance profiles. Also, despite the widespread reduction in H3K4me3, preserved levels of H3K4me3 were detected in certain genomic regions including the genes NBPF26, ACTN2, HRNR, and PLPRP5.
Software	Raw sequencing reads were first quality trimmed using Trim Galore 0.6.7.<br>Read mapping was performed with HISAT2 2.2.1 against the human genome (hg19) in paired-end mode.<br>Reads mapping to blacklisted regions were removed with the bedtools 2.30 intersect tool and sorted by coordinate and indexed using samtools 1.15.<br>Duplicated reads were removed using the MarkDuplicates tool from Picard.<br>BAM files were merged with samtools to create metagene H3k4me3 profiles with ngs.plot 2.61.<br>H3K4me3 peaks were identified using the MACS3 3.0.0.a6<br>H3K4 signal heatmap, PCA plot, sample distance heatmap and enriched pathway analysis of differential peaks was performed with R 4.2.0 using the Bioconductor packages DiffBind, ChIPseeker, and ReactomePA with prior annotation with HOMER 4.11.<br>Data was visualized with IGV.

Flow Cytometry

Plots

Confirm that:

☒ The axis labels state the marker and fluorochrome used (e.g. CD4-FITC).

☒ The axis scales are clearly visible. Include numbers along axes only for bottom left plot of group (a 'group' is an analysis of identical markers).

☒ All plots are contour plots with outliers or pseudocolor plots.

☒ A numerical value for number of cells or percentage (with statistics) is provided.

Methodology

Sample preparation	For human studies, neutrophils were stained with Zombie Aqua Fixable viability dye (Biolegend) for 15 min at room temperature to be able to exclude dead cells. Human Fc block (Biolegend) was used for 15 min at 4 degrees C to avoid unspecific subsequent antibody binding. Cells were next stained with an antibody mix (Table 2) for 30 min at 4 degrees C. FACS buffer (2 % v/v FBS in DPBS) was used as vehicle for Fc block and antibody mixes and for washes after staining. Stained neutrophils were fixed with 4 % PFA for 15 min on ice and washed before data acquisition.<br>For mouse studies, cells were harvested from the bone marrow and stained against CD16/32 (Biolegend) for 20 minutes followed by a lineage antibody cocktail 20 minute incubation. Linage positive events are removed by using magnetic beads and columns (Miltenyi) or directly based on fluorescence. A specific antibody cocktail was then used for 20 minutes followed by live/dead staining (Thermo Scientific) before running into the flow cytometer.

Instrument	BD LSRFortessa flow cytometer (Beckton Dickinson). BD FACSAria Fusion cell sorter.
Software	FlowJo (10.8.1) was used to analyse flow cytometry data.
Cell population abundance	Cell abundances are included in Extended Data Fig. 5.
Gating strategy	Human neutrophil counts: SSC-H/SSC-A> FSC-H/FSC-A> SSC-A/FSC-A (Beads and Granulocytes). Any eosinophils were excluded from the granulocytes gate based on their autofluorescent properties, as previously reported.<br>Human neutrophil phenotyping: SSC-H/SSC-A> FSC-H/FSC-A> SSC-A/Zombie Aqua>SSC-A/FSC-A (Cells)>CD66b+ CD49d->CD66b, CD16, and CD62L.<br>Ex vivo phagocytosis: SSC-H/SSC-A> FSC-H/FSC-A> SSC-A/FSC-A (Neutrophils)>CFS-E+<br>Mouse infection blood counts: SSC-H/SSC-A> FSC-H/FSC-A> SSC-A/Zombie Aqua> SSC-A/FSC-A> SSC-A/CD45+> SSC-A/Ly6G+<br>Cut&Run: SSC-A/FSC-A> SSC-H/SSC-A> FSC-H/FSC-A> Zombie Aqua/Lin-> cKit+/Sca1-> CD34+/CD16/32high (GMP) or CD34+/CD16/32low (CMP).<br>H3K4me3: SSC-A/FSC-A> SSC-H/SSC-A> FSC-H/FSC-A> SSC-A/Live/Dead-> Ly6G-/Lin-> CD16/32high/Ly6C+> cKithigh/CD34+> CD11blow/CD106- (ProNeu1) or CD11bhigh/CD106+ (ProNeu2). SSC-A/FSC-A> SSC-H/SSC-A> FSC-H/FSC-A> SSC-A/Live/Dead-> Ly6G-/Lin-> SiglecF-/CD11b+> cKit+/Gr1+> SSC-A /CD34- (PreNeu).<br>PreNeu sorting: SSC-A/FSC-A> SSC-H/SSC-A> FSC-H/FSC-A> SSC-A/Live/Dead-> Ly6G-/Lin-> SiglecF-/CD11b+> cKit+/Gr1+> SSC-A /CD34-<br>BCG vaccination study: SSC-A/FSC-A> SSC-H/SSC-A> FSC-H/FSC-A> SSC-A/Live/Dead-> Ly6G+

☒ Tick this box to confirm that a figure exemplifying the gating strategy is provided in the Supplementary Information.
