# Supplementary material for: Hypoxia induces histone clipping and H3K4me3 loss in neutrophil progenitors resulting in long-term impairment of neutrophil immunity
Source: Nat Immunol. 2025 Oct 28;26(11):1903–15. doi: 10.1038/s41590-025-02301-9 (PMC12571872; doi:10.1038/s41590-025-02301-9)
Supplement: Supplementary file 10 — Uncropped blot images with molecular weight markers. [file 41590_2025_2301_MOESM10_ESM.pdf]

Source images to Fig. 5e

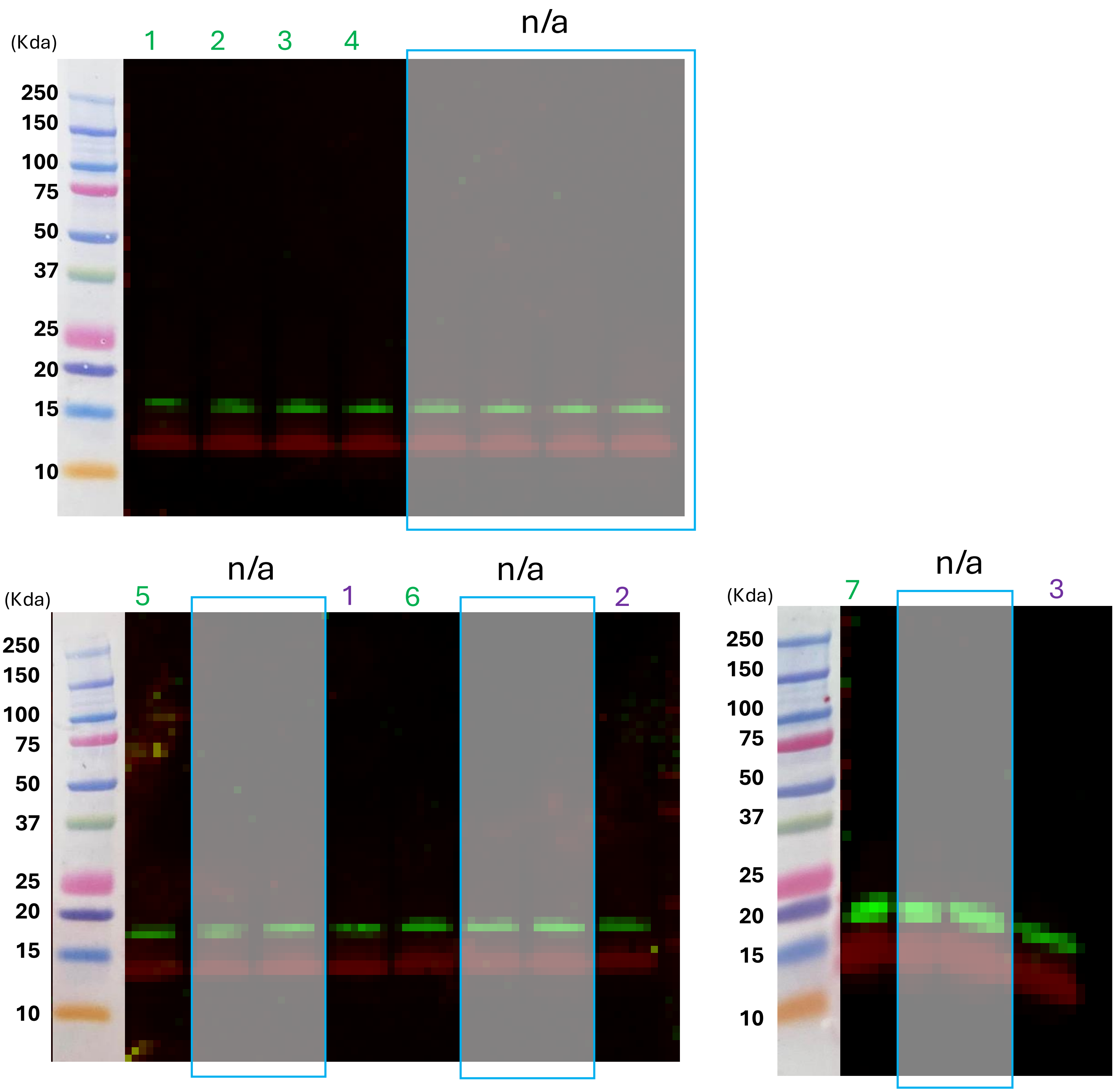

Blot images for the quantification shown in Fig. 5e. Each lane corresponds to an independent mouse sample. Green labels mark normoxia control mice and purple labels belong to hypoxic lung injury mice. n/a boxes are non-relevant samples run alongside within the same experiment. Antibody data and band sizes compiled in the table below.

Target	Primary antibody	Secondary antibody	Molecular weight
H3K4me3	Upstate 07-473 (rabbit)	Licor goat anti-rabbit 800CW (green band)	~17 KDa
H4	Abcam 31830	Licor goat anti-mouse 680RD (red band)	~ 11 KDa
